# Serum concentrations of BDNF in adolescents with metabolic syndrome: a case-control study between normal - BMI adolescents and adolescents with obesity

**DOI:** 10.1007/s00431-023-05129-3

**Published:** 2023-08-07

**Authors:** Flora Bacopoulou, Nikolaos G. Angelopoulos, Stavroula Papadodima, Despoina Apostolaki, Aimilia Mantzou, Eleni Koniari, Vasiliki Efthymiou, Artemis Tsitsika, Dimitrios Vlachakis, Evangelia Charmandari, Charikleia Stefanaki

**Affiliations:** 1 Center for Adolescent Medicine and UNESCO Chair in Adolescent Health Care, First Department of Pediatrics, Medical School, Aghia Sophia Children’s Hospital, National and Kapodistrian University of Athens, 11527 Athens, Greece; 2 University Research Institute of Maternal and Child Health and Precision Medicine, Medical School, National and Kapodistrian University of Athens, 11527 Athens, Greece; 3 Department of Forensic Medicine and Toxicology, Medical School, National and Kapodistrian University of Athens, 11527 Athens, Greece; 4 Unit of Clinical and Translational Research in Endocrinology, First Department of Pediatrics, Medical School, National and Kapodistrian University of Athens, Aghia Sophia Childrens Hospital, 1 Thivon Street, Goudi, 115 27 Athens, Greece; 5 MSc “Strategies of Developmental and Adolescent Health”, Second Department of Pediatrics, “P. & A. Kyriakou” Children’s Hospital, Medical School, National and Kapodistrian University of Athens, 11527 Athens, Greece; 6 Laboratory of Genetics, Department of Biotechnology, School of Applied Biology and Biotechnology, Agricultural University of Athens, 11855 Athens, Greece; 7 Division of Endocrinology, Metabolism and Diabetes, First Department of Pediatrics, Medical School, National and Kapodistrian University of Athens, Aghia Sophia Children’s Hospital, 11527 Athens, Greece; 8 Division of Endocrinology and Metabolism, Center for Clinical, Experimental Surgery and Translational Research, Biomedical Research Foundation of the Academy of Athens, 11527 Athens, Greece; 9 First Department of Pediatrics, National and Kapodistrian University of Athens, Thivon & Levadeias Str., Goudi, 11527 Athens, Greece

**Keywords:** Adolescents, Obesity, Brain derived neurotrophic factor, BDNF, Metabolic syndrome

## Abstract

Brain-Derived Neurotrophic Factor (BDNF) has been linked to various conditions of the cardiovascular and nervous systems. Scarce data exist about the concentrations of BDNF in children and adolescents in relation with obesity and metabolic syndrome (MetS). The aim of this study was to examine the serum BDNF concentrations in adolescents with metabolic syndrome and according to their body mass index (BMI) status. This was a case-control study, assessing BDNF concentrations between adolescents with MetS (with obesity vs. normal-BMI), in relation to sex, anthropometric, metabolic and endocrine parameters. Participants included male and female adolescents, whose anthropometric and metabolic panel, as well as serum BDNF concentrations were measured. A total of 59 adolescents (obesity: 29; normal-BMI: 30) were included in the study. Increased serum BDNF concentrations were observed in MetS adolescents with obesity when compared with normal-BMI adolescents (*p* < 0.001). Males exhibited higher concentrations of BDNF than females (*p* = 0.045). The sample was further divided into four categories by sex and BMI status, with normal-BMI females exhibiting significantly lower BDNF concentrations than females and males with obesity(*p* = 0.005). In the entire study sample, serum BDNF concentrations correlated positively with BMI z-scores, however, this statistical significance was preserved only in the females of the sample. No statistical difference was observed between males of different BMI z-scores categories.

*Conclusion*: Obesity appeared as a major factor for increased serum BDNF concentrations in adolescents with MetS (*vs.* normal-BMI), with a higher impact on BDNF concentrations in females than males.

**Table:** 

**What is Known:** *• The brain-derived neurotrophic factor (BDNF) is involved in metabolic syndrome in adults but data in adolescents are scarce.*	
**What is New:** *• Obesity (vs. normal BMI) was a major factor for increased serum BDNF in adolescents with metabolic syndrome.* *• Obesity had a higher impact on BDNF concentrations in females than males with metabolic syndrome.*

**What is Known:**

*• The brain-derived neurotrophic factor (BDNF) is involved in metabolic syndrome in adults but data in adolescents are scarce.*

**What is New:**

*• Obesity (vs. normal BMI) was a major factor for increased serum BDNF in adolescents with metabolic syndrome.*

*• Obesity had a higher impact on BDNF concentrations in females than males with metabolic syndrome.*

## Introduction

Brain-Derived Neurotrophic Factor (BDNF) is the most abundant neurotrophin in the central nervous system (CNS), especially in the hippocampus and cerebral cortex. Energy homeostasis centers within the hypothalamus, also, produce and secrete BDNF, as in other appetite-regulating areas including the vagal complex, hindbrain and ventral tegmental area of the midbrain. BDNF seems to increase insulin sensitivity, along with parasympathetic tone [1].

Genetic features, an unhealthy lifestyle, and stress disrupt BDNF signaling, possibly contributing to the pathogenesis of the metabolic syndrome. BDNF-focused interventions are currently under development for obesity, diabetes mellitus, and neurological disorders. It provokes extensive effects on the preservation of synaptic plasticity and promotion of new synapses in the hippocampus, where memory is acquired and solidified [2]. The BDNF gene is located on chromosome 11 and is expressed, not only, in the brain but, also, in other tissues, including human lymphocytes and monocytes. Of note, BDNF-producing haematopoietic cells control appetite and energy balance via migration to the hypothalamic paraventricular nucleus. These cells yield microglial markers and directly contact with neurons, regarding feeding status.

Circulating BDNF in the blood seems to be representative of its production in the brain, with most of it stored in platelets. Even if serum BDNF concentrations are multiple times higher than those in plasma, their correlation seems to be significant, and thus, their measurement seems to be interchangeable in many studies [3]. Decreased BDNF concentrations has been evidenced in many neurodegenerative and psychiatric conditions in adults, such as Alzheimer dementia [4], major depressive disorder [5], multiple sclerosis [6], Parkinson disease [7] and autism spectrum disorder [8]. All these disorders have been linked to decreased concentrations of BDNF in the brains of mammalian models, or in the blood serum of human adults [9]. An association of metabolic derangements and neurodegeneration has been evidenced [10], rendering BDNF as the culprit [11].

Several studies have reported low BDNF concentrations in adult patients with obesity [12], type 2 diabetes mellitus [13] and metabolic syndrome (MetS) [14]. Surprisingly, circulating BDNF concentrations in pediatric and adolescent patients were increased in autism spectrum disorders [15], decreased in Prader-Willi syndrome [16], increased in celiac disease [17], and similar to controls in children, adults and elderly with obesity [18]. Sexual dimorphism in the circulating concentrations of BDNF have, also, been studied, and sex hormones have been assumed to alter BDNF physiology [19]. Interestingly, studies about pediatric and adolescent populations with MetS in relation to BDNF are lacking.

The aim of this study was to examine fasting serum BDFN concentrations in adolescents with MetS, either with normal-body mass index (BMI) or with obesity and to explore potential associations of circulating BDNF concentrations with sex, anthropometric, metabolic, and endocrine parameters.

## Materials and methods

### Study design- setting

This case-control study took place at the Centre for Adolescent Medicine and UNESCO Chair in Adolescent Health Care (CAM) of the First Department of Pediatrics, National and Kapodistrian University of Athens, at the Aghia Sophia Children’s Hospital in Athens, Greece, from 01/01/2019 to 01/07/ 2019; (Ethics Committee protocol number 28126/09-12-15). Written consent was obtained from each adolescent and legal guardian, after thorough information about the purpose of the study.

### Participants

Post-pubertal adolescent males and females, aged 12–18 years, with an established diagnosis of MetS, who presented at CAM, were included. The diagnosis of MetS was based on the International Diabetes Federation criteria [20] and national waist circumference (WC) percentiles for abdominal obesity [21]. Male and female adolescents with MetS and obesity served as cases, whereas age-matched adolescents with MetS and normal-BMI served as controls. Overweight adolescents or those with diabetes mellitus, other severe comorbidities, chronic medication use, possible pregnancy, mental, developmental, genetic disorders, e.g. Down syndrome, were excluded.

### Definitions - procedures

MetS was diagnosed according to the International Diabetes Federation (IDF) criteria for adolescents (Table 1) [20].

**Table 1:** The IDF consensus definition of MetS in children and adolescents

**Age group**	**Abdominal Obesity (WC)**	**Triglycerides**	**HDL** **Cholesterol**	**Blood** **Pressure**	**Glucose**
10–16 years	WC ≥ 90^th^ percentile	≥ 1.7 mmol/L (≥ 150 mg/dL)	< 40 mg/dL	Systolic ≥ 130 Diastolic ≥ 85 mm Hg	≥ 5.6 mmol/L (100 mg/dL)
16 + years (same as adults)	WC ≥ 94 cm for males and ≥ 80 cm for females	≥ 1.7 mmol/L	< 40 mg/dL in males < 50 mg/dL in females	Systolic ≥ 130 Diastolic ≥ 85 mm Hg	≥ 5.6 mmol/L (100 mg/dL)

MetS diagnosis is defined by abdominal obesity plus any two of the remaining criteria

*IDF* International Diabetes Federation, *MetS* metabolic syndrome, *WC* waist circumference

 Participants had their morning blood samples collected, after an overnight fast. Hormonal and metabolic parameters including triglycerides, fasting glucose, and glycated hemoglobin (HbA_1c_) were analyzed immediately, using standard methodology. Supernatant serum was collected and stored at -80 °C until further analysis of the BDNF concentrations. Participants were also evaluated with a 2-h oral glucose tolerance test (OGTT). OGTT tests were performed after a 12 h fast, using 1.75 g/kg (maximum 75 g) of anhydrous glucose. Venous blood samples for glucose measurement were collected at 0, 30, 60, and 120 minutes [22, 23].

### Measurements

#### Anthropometry

All adolescents had their weight, height, waist circumference (WC) and hip circumference measured while barefoot and in light clothing. The weight and height were measured using an electronic scale with a stadiometer (Seca 217, Hamburg, Germany), while the waist and hip circumferences were measured using an inextensible anthropometric tape (Seca 201, Hamburg, Germany) twice with the adolescents standing erect and relaxed with arms at the sides and feet positioned close together. Waist circumference was measured midway between the lowest border of rib cage and the upper border of iliac crest, at the end of normal expiration. Hip circumference was measured at the widest part of the hip at the level of the greater trochanter. For all measurements the tape was positioned at a level parallel to the floor. All measurements were in centimeters (cm) rounded to the nearest 0.1 cm, and the ratios of waist-to-hip and waist-to-height were calculated for all participants. BMI was defined as the ratio of body weight to the square of height (kg/m^2^ [2]). All measurements were performed by the same designated physician. The WHO AnthroPlus software was used to determine the BMI-for-age z-score and percentile rank for each participant. BMI z-score cut-offs of 0 to 0.99 were considered as normal-BMI; 1 to 1.9 as overweight and ≥ 2.0 as obesity [24].

#### Systolic and diastolic blood pressure (BP)

The BP of all participants was measured using the DM-3000 device from Nissei Japan Precision Instruments. The systolic and diastolic BP were recorded in the right arm of each participant, in seated position and resting for a minimum of 10 min. Three consecutive measurements, separated by 5 min, were taken for each assessment. Mean arterial pressure (MAP) values were calculated for each participant using the systolic and diastolic BP readings. 

#### Blood parameters

Serum BDNF concentrations were measured by ELISA using the R&D Systems Quantikine ELISA kit. The sensitivity was 20 pg/mL, the intra-assay precision ranged from 3.8% to 6.2% and the inter-assay sensitivity ranged from 7.6% to 11.3%.

The OGTT interpretation and classifications were conducted according to the current guidelines. The OGTT results were analyzed using the area under the glucose curve (AUC_glucose_) and the insulin curve (AUC_insulin_), as estimated by the trapezoid rule. The AUC_glucose_ to AUC_insulin_ ratio was also calculated [25].

### Statistical analysis

*The p* values were calculated using 2-tailed tests, and statistical significance was established at *p* ≤ 0.05. The statistical analysis was performed using the SPSS software version 26 for Windows (IBM SPSS v.26.0). Further statistical analysis was conducted using R (R Core Team 2021). Continuous variables are presented as mean ± standard deviation (SD) or as median and interquartile range (IQR) based on their distribution normality. The Shapiro-Wilks test was employed for each parameter to evaluate for a statistically significant (*p*-value ≤ 0.05) difference of the data collected from normal distribution. For continuous data, the student’s t-test was used, or the Mann-Whitney U test for non-parametric data. Pearson or Spearman’s rho correlation coefficients were also calculated. Participants were further clustered into normal-BMI group and group with obesity, based on their BMI z-scores. Statistical significance between group measures was established using the Kruskal–Wallis rank sum.

### Study size

The study size of the sample was based on the calculations of Julius S.A. This was a pilot study, so 12 to 25 participants per group (Case group: adolescents with MetS and obesity; Control group: adolescents with MetS and normal-BMI) were considered as a appropriate numbers for statistical inference [26].

## Results

A total of 59 adolescents (mean age ± SD; 15.25 ± 1.8 years) diagnosed with MetS, agreed to participate in the study. Twenty-nine participants had obesity (mean age ± SD; 15.1 ± 1.9 years) whereas 30 adolescents presented with normal BMI (mean age ± SD; 15.4 ± 1.7 years). Among study participants, 31 were males [mean age ± SD; 15 ± 1.7 years, median BMI (IQR); 24.2 (12.1) kg/m^2^, and 28 were females [mean age ± SD; 15.5 ± 1.8 years, median BMI (IQR); 22.1 (11.5) kg/m^2^ (Table 2).

**Table 2:** Anthropometric, metabolic, and hormonal characteristics of the study sample

	**Total Sample** ***(n* = *59)***	**MetS adolescents with obesity** **(n = 29)**	**MetS adolescents with normal BMI** **(n = 30)**	***p***	**Females** ***(n* = *28)***	**Males** ***(n* = *31)***	***p***
Age (years)	15.2 ± 1.8	15.1 ± 1.92	15.4 ± 1.65	0.53	15.5 ± 1.8	15.0 ± 1.7	0.252
Serum BDNF (pg/mL)	21,089 ± 7,230	24,276 ± 5,425	18,009 ± 7,495	< 0.001^**^	19,110.5 ± 7,701.0	22,877.5 ± 6,383.1	0.045^*^
Height (cm)	168.4 ± 10.1	171 ± 8.2	166 ± 11.3	0.07	162.3 ± 6.7	173.9 ± 9.6	< 0.001^**^
Weight (kg)	72.1 ± 22.8	90.5 ± 16.5	54.3 ± 10.5	< 0.001^**^	64.5 ± 19.9	79.0 ± 23.4	0.014^*^
BMI (kg/m^2^)^§^	22.5 (11.5)	30.4 (5.6)	19.0 (3.7)	< 0.001^**^	22.1 (11.5)	24.2 (12.1)	0.335
BMI Percentile (%)^§^	84.4 (66.9)	99.5 (1.4)	34.3 (30.7)	< 0.001^**^	66.3 (70.26)	96.3 (51.3)	0.106
BMI z-score^§^	1.01 (3.04)	2.59 (0.79)	-0.41 (0.81)	< 0.001^**^	0.42 (3.01)	1.79 (2.79)	0.106
HbA1c (%)	5.1 ± 0.3	5.07 ± 0.3	5.1 ± 0.26	0.67	5.1 ± 0.3	5.1 ± 0.3	0.552
Triglycerides (mg/dL)^§^	87 (39)	106 (40)	76 (16)	< 0.001^**^	79 (35)	89 (38)	0.327
Fasting Glucose (mg/dL)	100.2 ± 9.4	99 ± 7.6	101 ± 10.8	0.36	100.1 ± 10.3	100.3 ± 8.8	0.934
Systolic BP (mmHg)	122.4 ± 16.9	133 ± 14.7	113 ± 12.4	< 0.001^**^	120.4 ± 15.3	124.1 ± 18.3	0.415
Diastolic BP (mmHg)	70.3 ± 12.6	75.7 ± 12.8	65 ± 10.2	< 0.001^**^	71.6 ± 13.3	69.2 ± 12.2	0.478
MAP (mmHg)	96.4 ± 13.5	104 ± 11.8	88.8 ± 10.3	< 0.001^**^	96.0 ± 13.4	96.6 ± 13.7	0.859
Fasting Insulin (mIU/L)^§^	11.2 (11.6)	8.4 (8.1)	11.2 (13.4)	0.46	14.2 (14.5)	5.4 (2.5)	**0.007**
Insulin AUC^a^ (mIU/L)^§^	192.8 (263.7)	409 (196)	149 (64.5)	0.002^*^	184.7 (124.5)	331.1 (299.6)	0.277
Glucose AUC^a^ (mg/dL)	480.6 ± 52.3	494 ± 41.6	465 ± 61.3	0.28	477.9 ± 59.7	483.8 ± 46.4	0.826
AUC_glucose_/AUC_insuline_^§^	2.23 (2.19)	1.4 (0.82)	3.02 (1.29)	0.002^**^	2.52 (1.65)	1.66 (2.22)	0.277
Waist Circumference (cm)	83.1 ± 17.6	91.7 ± 14	65.3 ± 8.4	< 0.001^**^	75.8 ± 18.6	88.8 ± 14.8	0.023^*^
Hip Circumference (cm)	102.7 ± 12.6	108 ± 10.4	89.1 ± 6.6	< 0.001^**^	101.8 ± 13.3	103.3 ± 12.5	0.740
Waist-to-Hip ratio^§^	0.81 (0.15)	0.87 (0.14)	0.73 (0.11)	0.03^*^	0.73 (0.08)	0.88 (0.11)	0.01^*^
Waist-to-Height ratio	0.49 ± 0.09	0.54 ± 0.07	0.39 ± 0.04	< 0.001^**^	0.46 ± 0.03	0.51 ± 0.02	0.140

Values are expressed as mean ± standard deviation (SD) or § median (interquartile range). The p-value was calculated using the t-test after the assumption of homogeneity of variance or § Mann-Whitney U test. Bold indicate statistically significant values

*MetS* Metabolic Syndrome, *BMI* Body Mass Index, *BDNF* Brain-Derived Neurotrophic Factor, *BP* Blood Pressure, *MAP* Mean Arterial Pressure

^a^AUC = Area Under the Curve from ground, calculated considering time = constant = 1

^*^Significance level < 0.05

^**^Significance level < 0.01

Serum BDNF concentrations differed significantly (*p* < 0.001) between participants with obesity (mean ± SD; 24,276 ± 5,425 pg/mL) and those with normal BMI (mean ± SD; 18,009 ± 7,495 pg/mL). Participants with obesity also demonstrated statistically significant higher measures of weight (*p* < 0.001), BMI (*p* < 0.001), BMI z-score (*p* < 0.001), triglycerides (*p* < 0.001), systolic, diastolic and mean arterial BP (*p* < 0.001), insulin AUC (*p* = 0.002), waist circumference (*p* < 0.001), hip circumference (*p* < 0.001), waist-to-hip ratio (*p* = 0.03), and waist-to-height ratio (*p* < 0.001).

In the total sample, male participants demonstrated statistically significant higher serum BDNF concentrations (*p* = 0.045), height (*p* < 0.001), weight (*p* = 0.014), fasting insulin (*p* = 0.007), waist circumference (*p* = 0.023) and waist-to-hip ratio (*p* = 0.01) than females.

**Fig. 1:**
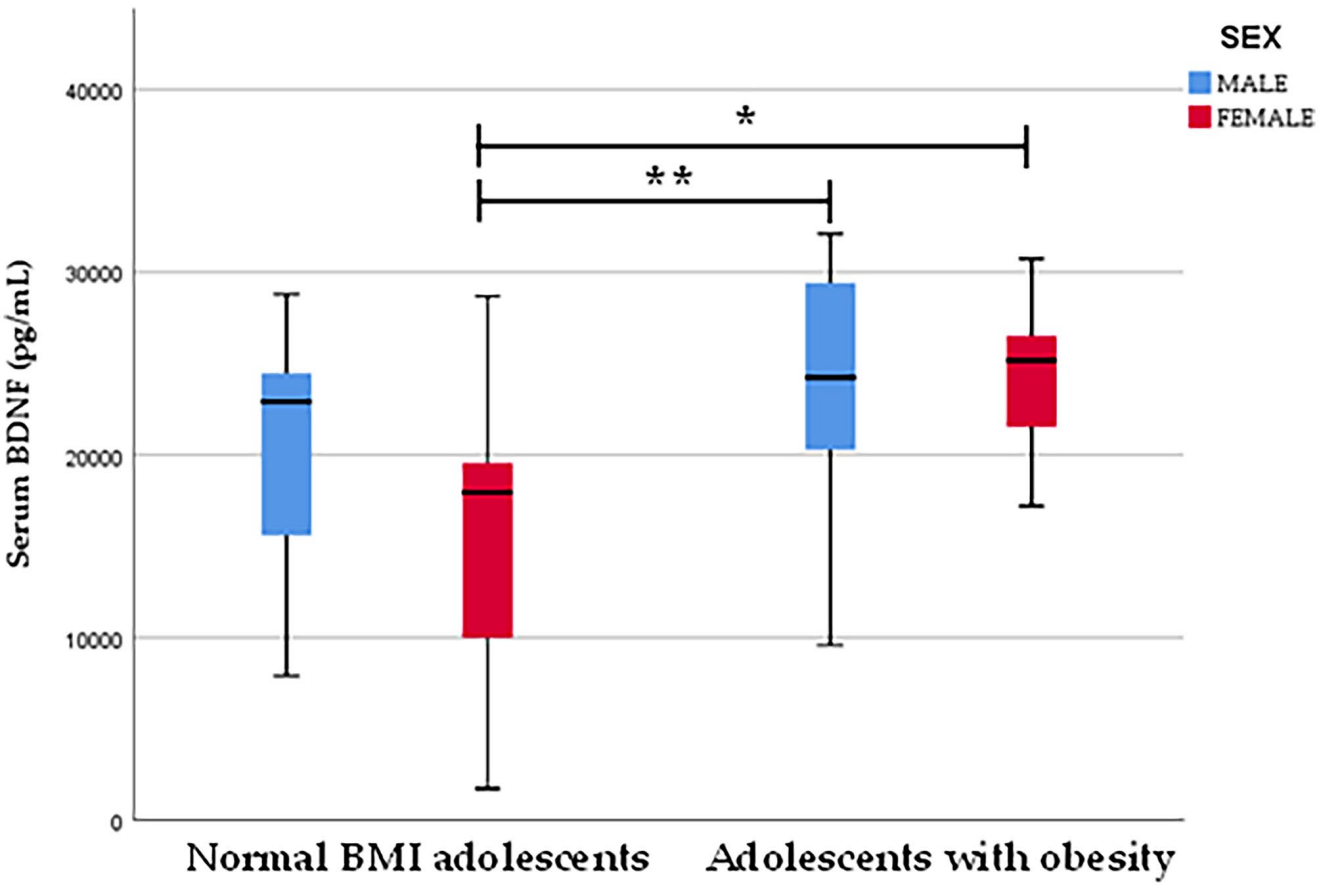
Differences in serum BDNF in adolescents with normal-BMI vs. obesity and according to sex. * *p *< 0.05, **p ≤ 0.01

Among female participants, mean BDNF concentration was significantly higher (*p* = 0.001) in those with obesity (mean ± SD; 24,235 ± 4,144 pg/mL) than females with normal-BMI (mean ± SD; 15,795 ± 7,721 pg/mL). Additionally, normal-BMI females and females with obesity demonstrated significant differences in weight (*p* < 0.001), systolic BP (*p* = 0.005), MAP (*p* = 0.006), waist circumference (*p* = 0.008) and waist-to-height ratio (*p* = 0.007). When comparing serum BDNF concentrations between males with obesity (mean ± SD; 24,301 ± 6,195 pg/mL) and normal-BMI males (mean ± SD; 20,906 ± 6,343 pg/mL), statistical significance was not detected (*p* = 0.147) (Fig. 1). Males with normal-BMI and those with obesity differed significantly in weight (*p* < 0.001), triglycerides (*p* = 0.02), systolic BP (*p* = 0.01), MAP (*p* = 0.02), waist circumference (*p* = 0.02), hip circumference (*p* = 0.04), and waist-to-height ratio (*p* = 0.016). There was no statistical difference in the age of participants between the sex and obesity status groups (Table 3).

**Table 3:** Sample characteristics by sex and BMI category

**Variables**	**Females with obesity** ***(n = 11)***	**Normal BMI Females** ***(n = 17)***	***p***	**Males with obesity** ***(n = 18)***	**Normal BMI Males** ***(n = 13)***	***p***
Age (years)	15.5 ± 2.0	15.6 ± 1.7	1	14.9 ± 1.9	15.1 ± 1.6	1
Serum BDNF (pg/mL)	24,235 ± 4,144	15,795 ± 7,721	**0.001** ^**^	24,301 ± 6,195	20,906 ± 6,343	0.147
Height (cm)	164 ± 5.9	161 ± 7.1	1	175 ± 6.6	172 ± 12.8	1
Weight (kg)	85.7 ± 12.0	50.8 ± 8.4	** < 0.001** ^**^	93.5 ± 18.4	58.9 ± 11.5	** < 0.001** ^**^
BMI z-score^§^	2.53 (0.73)	-0.5 (0.90)	** < 0.001** ^**^	2.63 (0.89)	-0.2 (0.66)	** < 0.001** ^**^
Triglycerides (mg/dL)^§^	106 (44)	75.5 (18)	0.17	108 (29)	76 (13)	**0.02** ^*^
Systolic BP (mmHg)	134 ± 11.9	112 ± 10.8	**0.005** ^**^	132 ± 16.4	113 ± 14.6	**0.01** ^*^
Diastolic BP (mmHg)	81 ± 13.9	65.7 ± 9.1	0.11	72.8 ± 11.5	64.2 ± 11.7	0.28
MAP (mmHg)	107 ± 11.8	89 ± 9.0	**0.006** ^**^	103 ± 11.9	88.5 ± 12.1	**0.02** ^*^
Fasting Glucose (mg/dL)	96.6 ± 5.7	102 ± 11.7	1	100. ± 8.2	101 ± 10	1
Fasting Insulin (mIU/L)^§^	14.2 (10.1)	14.3 (13)	1	5.26 (3.56)	5.45 (1.6)	1
Insulin AUC^a^ (mIU/L)^§^	324. (182)	171 (41.7)	0.84	409 (148)	119 (4.4)	0.52
Waist Circumference (cm)	87.6 ± 16.5	60.6 ± 4.9	**0.008** ^**^	94 ± 12.4	72 ± 8	**0.02** ^*^
Hip Circumference (cm)	107 ± 11.2	89.5 ± 9.3	0.16	108 ± 10.2	88.8 ± 4.6	**0.04** ^*^
Waist-to-Hip ratio^§^	0.73 (0.08)	0.68 (0.08)	0.23	0.88 (0.04)	0.81 (0.04)	0.39
Waist-to-Height ratio	0.52 ± 0.09	0.37 ± 0.04	**0.007** ^**^	0.54 ± 0.06	0.40 ± 0.05	**0.016** ^*^

Values are expressed as mean ± standard deviation (SD) or § median (interquartile range). The p-value was calculated using the t-test after the assumption of homogeneity of variance or § Mann-Whitney U test. Bold indicate statistically significant values

*BMI* Body Mass Index, *BDNF* Brain-Derived Neurotrophic Factor, *BP* Blood Pressure, *MAP* Mean Arterial Pressure

*Significance level < 0.05

**Significance level < 0.01

^a^AUC = Area Under the Curve from ground, calculated considering time = constant = 1

Amongst all participants, serum BDNF concentrations were positively correlated with height (r_s_ = 0.32, *p* = 0.012), weight (r_s_ = 0.44, *p* = 0.001), BMI (r_s_ = 0.37, *p* = 0.004), BMI z-score (r_s_ = 0.39, *p* = 0.002) and systolic BP (r_s_ = 0.29, *p* = 0.028). Among female participants, serum BDNF concentrations were positively correlated with weight (r_s_ = 0.51, *p* = 0.006), BMI (r_s_ = 0.44, *p* = 0.020), BMI z-score (r_s_ = 0.50, *p* = 0.007), and systolic BP (r_s_ = 0.50, *p* = 0.009). Among male participants, no significant correlations between serum BDNF concentrations and the examined parameters were observed (Table 4).

**Table 4:** Statistically significant Spearman rho correlation coefficients between serum BDNF concentrations and study sample anthropometric characteristics

**Variables**	**Total sample**		**Females**		**Males**	
	**r**_**s**_	***p***	**r**_**s**_	***p***	**r**_**s**_	***p***
Height (cm)	0.32*	**0.012**	0.31	0.105	0.13	0.491
Weight (kg)	0.44**	**0.001**	0.51**	**0.006**	0.24	0.192
BMI (kg/m^2^)	0.37**	**0.004**	0.44*	**0.020**	0.25	0.174
BMI z-score	0.39**	**0.002**	0.50**	**0.007**	0.24	0.190
Systolic BP (mmHg)	0.29*	**0.028**	0.50**	**0.009**	0.08	0.668

Bold indicate statistically significant values

*BDNF* Brain-Derived Neurotrophic Factor, *BMI* Body Mass Index, *BP* Blood Pressure

*Correlation significant at the 0.05 level

**Correlation significant at the 0.01 level

## Discussion

In this study, we examined serum BDNF concentrations between adolescents with MetS (with normal-BMI *vs.* obesity. Adolescents with MetS and obesity presented with increased BDNF concentrations when compared with their normal-BMI counterparts. Overall, male participants exhibited significantly greater serum BDNF concentrations than their female counterparts, while failing to demonstrate significant correlations with anthropometric, metabolic, and endocrine parameters. Normal-BMI females had significantly lower BDNF concentrations, when compared with females and males with obesity. Additionally, this study demonstrated positive correlations in the whole sample between serum BDNF concentrations, systolic BP, body weight, BMI, and BMI z-score. In the females only, BMI z-scores demonstrated strong, positive, statistically significant associations with BDNF concentrations. A positive correlation was, also, demonstraded between systolic BP and serum BDNF in females. 

To our knowledge, this is the first study, assessing BDNF concentrations in relation to anthropometric, metabolic, and endocrine parameters in adolescents with MetS, assessing, also, the sex, and BMI status. Iughetti et al. found a significant negative correlation with BMI, by measuring plasma BDNF concentrations in children and adolescents [27]. In adults, Lommatzsch et al. reported a significant difference between healthy, females and males, regarding their platelet BDNF. Specifically, platelet BDNF concentrations in females were lower than males and changed along the menstrual cycle [28]. Also, Lommatzsch et al. found a negative correlation between plasma BDNF and body weight [28]. On the other hand, a meta-analysis of ten studies demonstrated similar circulating BDNF levels in obese patients and controls [18].

In our study, obesity had minor, or no impact on serum BDNF concentrations in males, as opposed to females. Previous studies, reporting differences between the two sexes in either serum, or plasma BDNF concentrations, are in line with the findings of this study. Lee et al. reported no difference in fasting serum BDNF concentrations between males with obesity and normal-BMI [29], while, Guzel et al. demonstrated significantly lower BDNF concentrations in adult women with obesity than normal weight females [30]. Gajewska et al. studied adults 45 to 86 years of age, with obesity and normal body weight, and found that sex, age, and obesity did not influence serum concentrations of BDNF [31].

Obesity, an indispensable element of MetS, has consistently been identified as a risk factor for neurodegenerative and psychiatrics disorders. Research has linked the dysregulation of the BDNF gene to, both, obesity and diseases of the CNS [32]. Also, these results seem to support the hypothesis of a potential pathophysiologic mechanism of BDNF in obesity. BDNF is implicated in energy homeostasis by promoting the feeling of satiety, indirectly downregulating the signaling pathway of leptin, suggesting that the upregulation of BDNF acts as a response to increased adiposity [33].

Similarly to obesity, there are several other conditions in pediatric and adolescent populations, in which increased BDNF concentrations have been reported in comparison with healthy controls. Drug-naïve pediatric patients with attention deficit - hyperactivity disorder (ADHD) [34], children with autism spectrum disorder (ASD) [15], and adolescents with major depression (MDD) [35] have demonstrated increased concentrations of BDNF than healthy controls. This increase of BDNF concentrations is believed to have a neuroprotective role in the developing brain. Remarkably, adults with ADHD [36] or ASD [8] presented with significantly lower BDNF concentrations than healthy controls. These observations suggest that, at some point, the upregulation of BDNF that feasibly acts as a compensatory mechanism, comes to a halt. The same speculation may be applied in celiac disease, where adult patients have lower serum BDNF concentrations [37], whereas children with celiac disease have increased BDNF concentrations in comparison with healthy controls [17].

Adults with obesity have low BDNF concentrations in serum [11], while adolescents with obesity in this study demonstrated an increase. In female adolescents, obesity may activate the compensatory mechanism of BDNF upregulation, causing higher concentrations, whereas chronic obesity can lead to inadequate BDNF production, comparably evidenced in other disorders with BDNF upregulation. Additionally, there are some data, supporting upregulation of BDNF production by the estrogens [38].

The relatively small number of participants with MetS could be regarded as a limitation of this study. However, given the low prevalence of MetS in adolescents [39], the study sample can be considered representative of the Greek population under study. MetS is considered an infrequent disease in adolescence in Greece, rendering the nature of this study ideal [40]. 

Confounding factors, such as pubertal status, menstrual phase, time of blood sampling, fasting state, smoking status and platelet count were taken into consideration [18]. In this study, BDNF concentrations were measured in frozen preserved blood serum samples. Various studies report measurements of either plasma or serum BDNF, with the former being lower than the latter, and both being positively correlated to each other and the platelet count. In addition, BDNF concentrations in blood seem to represent adequately those at the CNS, due to the established positive correlation between brain and circulating BDNF. Existing evidence argues intact BDNF in the peripheral circulation is transported through the blood-brain barrier. Peripherally synthesized BDNF has also been observed, as in vascular endothelial cells, however its importance in the regulation of BDNF concentrations in the CNS is not fully understood. Circulating plasma BDNF is considered biologically active, however it is more susceptible to external factors than serum, hence, assessing BDNF concentrations in coagulated blood serum may be better suited for research and clinical purposes.

The results of the present study indicate the distinct effect of obesity and sex on BDNF concentrations during adolescence and provide hints for the development of more personalized interventions and ways of prevention. It has already been demonstrated that physical activity and exercise interventions have different effects on male and female individuals, with males showing overall greater changes in BDNF concentrations than females [41], while females experience changes in BDNF concentrations primarily through diet intervention [42]. Studies on the physiology of BDNF may explain the link between obesity, MetS and neurodegenerative disorders [11], as well as the potential for early prevention, diagnosis, and novel treatments [1].

Future research should include larger samples and more sophisticated design in pediatric and adolescent populations to elaborate on the physiology of BDNF regarding metabolic disorders. Lastly, the hypothesis concerning the compensatory upregulation of BDNF in adolescents with obesity and MetS needs to be further explored, and examined in other early disruptive processes that carry on to adulthood.

## Conclusions

In the present study obesity appeared as a major factor for increased serum BDNF concentrations in adolescents with MetS (*vs.* normal BMI), with a higher impact on BDNF concentrations in females than males. These findings seem to form a basis for further study of the BDNF physiology and lend support to a novel hypothesis regarding BDNF upregulation in adolescence.

## Data Availability

Data are available upon request.
